# A Novel Quinoline Inhibitor of the Canonical NF-κB Transcription Factor Pathway

**DOI:** 10.3390/biology13110910

**Published:** 2024-11-07

**Authors:** Panagiotis Ntavaroukas, Konstantinos Michail, Rafaela Tsiakalidou, Eleni Stampouloglou, Katerina Tsiggene, Dimitrios Komiotis, Nikitas Georgiou, Thomas Mavromoustakos, Stella Manta, Danielle Aje, Panagiotis Michael, Barry J. Campbell, Stamatia Papoutsopoulou

**Affiliations:** 1 Department of Biochemistry and Biotechnology, School of Health Sciences, University of Thessaly, 41335 Larissa, Greece; davapanos@gmail.com (P.N.); kostantinemix@gmail.com (K.M.); raf_tsiakalidou@hotmail.com (R.T.); elenhstampouloglou@gmail.com (E.S.); ktsiggene@gmail.com (K.T.); dkom@uth.gr (D.K.); smanta1@yahoo.gr (S.M.); 2 Laboratory of Organic Chemistry, Department of Chemistry, National and Kapodistrian University of Athens, Panepistimioupolis Zografou, 11571 Athens, Greece; nikitage@chem.uoa.gr (N.G.); tmavrom@chem.uoa.gr (T.M.); 3 Laboratory of Organic Chemistry, Faculty of Chemistry, Aristotle University of Thessaloniki, 54124 Thessaloniki, Greece; 4 The Henry Wellcome Laboratories of Molecular & Cellular Gastroenterology, Department of Infection Biology & Microbiomes, Institute of Infection Veterinary and Ecological Sciences, University of Liverpool, Liverpool L69 3BX, UK; d.aje@liverpool.ac.uk (D.A.); panagiotis.a.michael@gmail.com (P.M.); 5 Department of Molecular Biology and Genetics, Democritus University of Thrace, 68100 Alexandroupolis, Greece

**Keywords:** NF-κB, inflammation, quinoline, transcription

## Abstract

The NF-κB family of transcription factors is a master regulator of cellular responses during inflammation, and its dysregulation has been linked to chronic inflammatory diseases, such as inflammatory bowel disease. It is therefore of vital importance to design and test new effective NF-κB inhibitors that have the potential to be utilized in clinical practice. In this study, we used a commercial transgenic HeLa cell line as an NF-κB activation reporter to test a novel quinoline molecule, Q3, as a potential inhibitor of the canonical NF-κB pathway. Q3 inhibited NF-κB-induced luciferase in concentrations as low as 5 μM and did not interfere with cell survival or induced cell death. A real-time PCR analysis revealed that Q3 could inhibit the TNF-induced transcription of the luciferase gene, as well as the *TNF* gene, a known downstream target gene. Immunocytochemistry studies revealed that Q3 moderately interferes with TNF-induced NF-κB nuclear translocation. Moreover, docking and molecular dynamics analyses confirmed that Q3 could potentially modulate transcriptional activity by inhibiting the interaction of NF-κB and DNA. Therefore, Q3 could be potentially developed for further in vivo studies as an NF-κB inhibitor.

## Simple Summary

Many acute and chronic diseases, including cancer, are characterized by the dysregulation of specific molecules that regulate gene expression and belong to the NF-κB family. It is therefore vital to design and experimentally test new inhibitors that have the potential to be utilized in vivo. Here, we present a novel quinoline molecule Q3 that was tested on an NF-κB reporter cell line and has the potential to inhibit the function of NF-κB. In the presence of Q3, we observed reduced signals in TNF-activated reporter cells, as well as a reduced transcription of specific NF-κB target genes. Moreover, a computer-based analysis revealed that the Q3 molecule potentially interferes with the DNA-binding activity of the transcription factor tested and therefore would be developed in a specific inhibitor of the NF-κB pathway.

## 1. Introduction

The nuclear factor kappa-light-chain-enhancer of activated B cells (NF-κB) family of transcription factors comprises homo- or heterodimers formed by five structurally related proteins, namely RelA (p65), RelB, cRel, p105/p50 (NF-κB1), and p100/p52 (NF-κB2) [1]. Whilst certain dimeric complexes are restricted to specific cell types, the p50/p65 heterodimer is the most widespread and predominant active form of NF-κB, found in nearly all cells [2]. NF-κB activity is typically inducible and can be triggered by a broad range of extracellular stimuli through two main signaling pathways, the canonical and non-canonical pathways [3], that interact with each other but modulate different signaling molecules and NF-κB dimers, therefore regulating different target genes. Under resting conditions, NF-κB dimers are sequestered in the cytoplasm by inhibitors of NF-κB (IκB) proteins. Upon stimulation, signaling cascades involving various adaptor proteins and kinases are activated, leading to the phosphorylation, polyubiquitination and subsequent degradation of IκB proteins by the proteasome. This allows NF-κB dimers to translocate into the nucleus, where they activate the transcription of target genes [4]. Given their role in regulating genes associated with critical cellular processes, including immune responses, proliferation, survival, and apoptosis, the dysregulation of NF-κB signaling can lead to serious pathologies such as immunodeficiency, autoimmunity, and cancer [5,6,7]. The canonical NF-κB pathway is a major regulator of immune cells, driving inflammatory responses in both innate and adaptive compartments [8]. Aberrant NF-κB activation has been observed in intestinal mucosal biopsies from inflammatory bowel disease (IBD) patients with active Crohn’s disease or ulcerative colitis, promoting the production of pro-inflammatory mediators [9,10]. Elevated levels of NF-κB p65 subunit proteins or a high DNA-binding activity of NF-κB has been detected in macrophages and epithelial cells isolated from inflamed intestinal biopsies of IBD patients [11,12,13,14]. Some of these mediators further enhance NF-κB activity, creating a positive feedback loop that perpetuates the disease pathology [13].

A deep understanding of the molecular mechanisms underlying NF-κB activation has positioned this transcription factor as a promising therapeutic target due to its pivotal role in inflammatory responses [15]. Numerous inhibitors targeting this pathway have been experimentally identified, ranging from broad-spectrum agents to more selective inhibitors [16,17,18]. Some of these have progressed to clinical use, including both natural and synthetic molecules [19,20]. Various commonly used anti-inflammatory drugs modulate NF-κB activation as part of their therapeutic action, including glucocorticoids, non-steroidal micromolecular compounds, monoclonal antibodies, and small interfering RNAs (siRNAs) [21,22]. However, a subset of patients either do not respond or they experience adverse side effects from these treatments [23]. Amongst the drug classes, small-molecule inhibitors hold significant promise in drug development due to their ease of design, synthesis, and modification [24].

Quinolines and quinolones, synthesized through structural modifications of quinine, are traditionally recognized for their antimicrobial properties [25], where they offer significant potential as therapeutic agents due to their ability to be easily modified by attaching various substituents to their pharmacophoric core, thereby expanding their versatility and enhancing their therapeutic efficacy. They act by inhibiting bacterial DNA gyrase or topoisomerase IV [26,27]. This inhibition disturbs DNA stability, ultimately leading to bacterial death [28]. Most of the research undertaken on quinolines and quinolones is focused on addressing the problem of microbial antibiotic resistance, with multiple attempts to synthesize new, more sophisticated, and capable antimicrobial compounds [26]. These are versatile compounds with anti-malarial, anti-bacterial, anti-trypanosomal, and anti-tuberculosis activity [29]. They have also been reported to inhibit NF-κB activation, mainly in in vitro models [30]. Fluoroquinolone antibiotics such as ciprofloxacin and levofloxacin were reported to inhibit the microglia inflammatory response mediated by NF-κB downstream Toll-like receptor 4 (TLR4) signaling [31]. Additionally, moxifloxacin was shown to inactivate mitogen-activated protein kinases (MAPKs) ERK1/2 and p38, the p65-NF-κB signaling pathway, and pro-inflammatory cytokine synthesis in human peripheral blood monocytes infected with *Aspergillus fumigatus* [32]. Decoy NF-κB nucleotides bind with the p65 Rel homology domain (RHD), which further inhibits the interaction of the p65 subunit with cis-elements in the DNA. At present, though, no small molecule has yet been developed that can modify interactions between p65/NF-κB and DNA [24]. Hence, there is a need to develop new compounds that have a stronger affinity to pro-inflammatory NF-κB dimers.

In this study, we evaluated in vitro the effect of a novel quinoline as an inhibitor of the canonical NF-κB pathway using a luciferase reporter human epithelial cell line. We showed that the specific quinoline does not inhibit TNF-induced IκB degradation and release of the NF-κB dimers in the cytoplasm, but it does inhibit the transcription of known NF-κB target genes, including the luciferase transgene. Moreover, an in silico analysis revealed that this quinoline, Q3, potentially interfered with the DNA-binding activity of the p65/NF-κB transcription factor, whereas the control molecule, Q1, did not have any effect.

## 2. Materials and Methods

### 2.1. Quinolines

**Figure 1:**
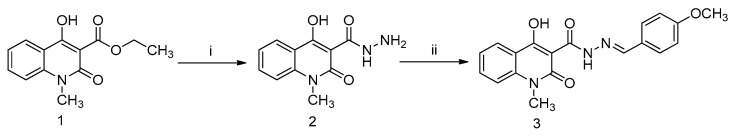
Schematic representation of the structure and synthesis of the novel Q3 quinoline. Reactions of (i) hydrazine and methanol for 30 min at 100 °C; and (ii) 4-methoxybenzaldehyde, *o*-phosphoric acid, and DMF/ethanol (1:1) for 6 h at reflux temperature. Compounds (1) ethyl 4-hydroxy-1-methyl-2-oxo-1,2-dihydroquinoline-3-carboxylate (Q1); (2) carbohydrazide intermediate compound; and (3) 4-hydroxy-N’-(4-methoxybenzylidene)-1-methyl-2-oxo-1,2-dihydroquinoline-3-carbohydrazide (Q3).

The novel quinolines were synthesized as part of this study in the laboratory of BioOrganic Chemistry, Department of Biochemistry and Biotechnology, University of Thessaly. The structures of the synthesized quinoline molecules are presented in Figure 1. All quinoline stock solutions were solubilized in dimethyl sulfoxide (DMSO) to a final concentration of 100 mM and stored at room temperature.

#### 2.1.1. General Methods

Melting points were recorded in a DigiMelt MPA 160 Mel-Temp apparatus (Stanford Research Systems, Sunnyvale, CA, USA) and are uncorrected. Thin-layer chromatography (TLC) was performed on Merck pre-coated 60F254 plates (Merck; Darmstadt, Germany). Reactions were monitored by TLC on silica gel, with detection by UV light (254 nm) or by charring with sulfuric acid. Flash column chromatography was performed using silica gel (240–400 mesh; Merck). ^1^H and ^13^C NMR spectra were obtained at ambient temperature using a Bruker Avance 500 spectrometer at 500 and 125 MHz, respectively, using chloroform-*d* (CDCl_3_) with internal tetramethylsilane (TMS). Chemical shifts (δ) are given in ppm measured downfield from TMS and spin–spin coupling constants in Hz. Mass spectra were obtained on a ThermoQuestFinnigan AQA Mass Spectrometer (electrospray ionization) (Thermo Fisher Scientific; Waltham, MA 02451, USA). All reactions sensitive to oxygen or moisture were carried out under an argon atmosphere.

#### 2.1.2. Synthesis of the Novel Q3 Quinoline

Quinoline compound **1** was synthesized in the lab as previously reported [33]. A reaction of the well-known ethyl 4-hydroxy-1-methyl-2-oxo-1,2-dihydroquinoline-3-carboxylate (quinoline compound **1**; Q1) with hydrazine hydrate provided the corresponding carbohydrazide intermediate compound **2**. Compound **2** interacted with 4-methoxy benzaldehyde in dimethyl formamide (DMF)/ethanol with a catalytic amount of *o*-phosphoric acid, with the aim of producing the target compound 4-hydroxy-*N’*-(4-methoxybenzylidene)-1-methyl-2-oxo-1,2-dihydroquinoline-3-carbohydrazide (quinoline compound **3**; Q3). A solution of compound **2** (0.23 g, 1.0 mmol) in DMF/ethanol (1:1, 10 mL) was prepared, and two drops of phosphoric acid were then added to the solution. After that, the reaction continued by adding 4-methoxy benzaldehyde (0.18 mL, 1.5 mmol) to the mixture and refluxed for 6 h. When the reaction was completed (monitored with TLC), the reaction temperature was lowered in an ice bath.

### 2.2. Tissue Culture and Reagents

The human NF-κB luciferase reporter HeLa stable cell line (HeLa/NF-κB-Luc; SL-0001—Signosis, Santa Clara, CA, USA) that expresses inducible luciferase under the promoter of the canonical NF-κB pathway was used for the study. After initial selection over 3 days using 50 μg/mL hygromycin B (#10687010 —Invitrogen; Paisley, UK), cultured cells were routinely maintained in DMEM, 10% (*v*/*v*) fetal bovine serum (FBS), 10 U/mL penicillin, 10 mg/mL streptomycin, and 2 mM L-glutamine (all Sigma-Aldrich; Poole, UK) in a humidified incubator at 37 °C in an atmosphere of 5% CO_2_. For passaging, cells were detached using 0.05% (*w*/*v*) Trypsin–EDTA (Gibco; Paisley, UK) for 5 min at 37 °C and counted using either a Neubauer cell counting chamber (Sigma-Aldrich, Poole, UK) or a TC10™ automated cell counter (Bio-Rad, Hemel Hempstead, UK). Cells in suspension were then re-seeded to flasks, either for maintenance or to plates for experimentation. Human recombinant tumor necrosis factor (TNF; PeproTech; London, UK) was used to stimulate the HeLa/NF-κB-Luc cells at 20 ng/mL. Known NF-κB pathway inhibitors used included the proteasome inhibitor MG132 (Selleckchem; Cambridge, UK) [28,34,35], the macrolide antibiotic clarithromycin [Maxilin] (Anfarm Hellas S.A.; Athens, Greece) [36,37], and hydrocortisone (Sigma-Aldrich, Poole, UK) [27,38,39].

Three additional intestinal cell lines, obtained from the American Type Culture Collection (ATCC—LG Standards; Teddington, UK), were seeded at 1 × 10^5^ cells/well in 24-well plates (Greiner CELLSTAR flat bottom; Sigma-Aldrich, Poole, UK) and used to further examine the action of the quinoline Q3 on TNF-induced *TNF* expression. Colorectal HT29 (ATCC—HTB-38) and DLD-1 (ATCC—CCL-221) cells, as well as Int-407 cells (ATCC—CCL-6), were maintained in complete medium as per ATCC recommendations.

### 2.3. Luciferase Reporter Assay

HeLa/NF-κB-Luc cells were cultured in 100 μL medium in 96-well plates (Greiner CELLSTAR flat bottom; Sigma-Aldrich, Poole, UK) and incubated overnight. The next day, the cell cultures were left untreated or pretreated with inhibitors for 20 min prior to stimulation with TNF in a final volume of 200 μL. Initial experiments were performed to define the conditions for the luciferase assay, where the number of cells seeded were 0.5 × 10^4^ to 2 × 10^4^ cells/well and the TNF dose and treatment time ranged from 10 to 40 ng/mL and 1 to 3 h, respectively. At the end of the stimulation, cells were washed once with phosphate-buffered saline (PBS) at pH 7.3 (Gibco), and luciferase activity was measured following cell lysis using a Bright-Glo kit (E2620—Promega; Southampton, UK), as per the manufacturer’s instructions. Cell lysates were transferred to 96-well white-walled plates (Nunc-Immuno microwell P8616; Sigma-Aldrich, Poole, UK), and luminescence was detected in an EnSpire multimode plate reader (PerkinElmer Inc., Waltham, MA, USA) or an Infinite F200 plate reader (Tecan; Reading, UK).

### 2.4. Viability Assay

HeLa/NF-κB-Luc cells were cultured in 12-well plates (Greiner CELLSTAR; Sigma-Aldrich, Poole, UK) at 0.5 × 10^6^ cells/well in 2 mL medium and incubated overnight. The next day, they were either left untreated or they were stimulated with 20 ng/mL TNF for 3 h. At the end of the stimulation, the cells were washed with cold PBS, scraped, and resuspended in 100 μL PBS. Cells were stained with 7-amino actinomycin D (7AAD), according to manufacturer guidelines (Invitrogen) and analyzed by flow cytometry using a Cytomics FC 500 Beckman Coulter flow cytometer (Beckman Coulter, Inc.; Fullerton CA, USA). Raw data analysis was performed using CXP software 2.0 (Beckman Coulter).

### 2.5. Western Blot

HeLa/NF-κB-Luc cells were cultured at 1 × 10^6^ cells/well in 2 mL medium in 6-well culture plates (Greiner CELLSTAR; Sigma-Aldrich). They were either left unstimulated or they were stimulated with 20 ng/mL TNF in the absence or presence of 40 μM MG132, 5 μM Q1, or 5 μM Q3 for 20 min. At the end of the stimulation, the cells were washed in ice-cold PBS and lysed in 150 μL radioimmunoprecipitation assay (RIPA) buffer (Sigma-Aldrich) for 20 min on ice, followed by centrifugation at 10,000 x *g* for 10 min. The supernatant was mixed with 4× Laemmli sample buffer (Bio-Rad; Hemel Hempstead, UK) and boiled for 3 min at 95 °C. SDS-PAGE was carried out using Mini-Protean TGX pre-cast gels (Bio-Rad, Hemel Hempstead, UK) on a Mini-Protean II gel electrophoresis system (Bio-Rad, Hemel Hempstead, UK) with pre-stained SDS-PAGE standards as molecular weight markers (161-0318; Bio-Rad, Hemel Hempstead, UK). Proteins were transferred to methanol-activated polyvinylidene difluoride (PVDF) membranes (Thermo Fisher Scientific; Horsham, UK) using a Mini Trans-Blot Cell Gel Electrophoresis System (Bio-Rad, Hemel Hempstead, UK) for 1 h at 400 mA. Membranes were subsequently blocked with PBS containing 0.05% (*v*/*v*) Tween-20 and 5% *w*/*v* skim milk powder for 1 h at room temperature, followed by primary antibody incubation overnight at 4 °C. Membranes were washed twice with PBS 0.05% (*v*/*v*) Tween-20 and were incubated with a secondary antibody for 1 h at room temperature. This was followed by two washes with PBS 0.05% (*v*/*v*) Tween-20. Proteins were detected by enhanced chemiluminescence (ECL) using a SuperSignal Western blot enhancer (#334095; Thermo Fisher Scientific, Waltham, MA 02451, USA) and visualized with the Molecular Imager Gel Doc XR System (Bio-Rad, Hemel Hempstead, UK). The band density was quantified by ImageLab version 3.0.1 (Bio-Rad, Hemel Hempstead, UK).

The following primary antibodies were used: rabbit anti-IκBα (#44D4 at 1:1000 dilution) and rabbit anti-β tubulin (#2146 at 1:1000 dilution), both obtained from Cell Signaling Technology Inc. (Danvers, MA, USA). Membranes were first probed with the anti-IκBα antibody following chemiluminescent detection and densitometry analyses. They were then rinsed in PBS and subsequently re-probed with the anti-β tubulin antibody to quantify loading. The secondary anti-rabbit immunoglobulin (IgG) horse radish peroxidase (HRP) conjugate antibody (#7074—Cell Signaling Technology Inc.) was used at a dilution of 1:2000.

### 2.6. Immunocytochemistry

Immunocytochemistry was performed in Falcon 8-well CultureSlides (Corning; New York, NY, USA) precoated with 300 μL of 0.1 mg/mL Poly-D-Lysine (Gibco; Paisley, UK) for 1 h at room temperature and air dried for 1 h with an open lid inside the hood. HeLa/NF-κB-Luc cells were cultured at 3 × 10^4^ cells/well in 0.3 mL Dulbecco’s Modified Eagle Medium (DMEM GlutaMAX) containing 10% *v*/*v* fetal calf serum (FCS) and 1% (*v*/*v*) penicillin/streptomycin (all Gibco) and left to rest overnight. The next day, the cells were left untreated or were pretreated for 30 min with either 40 μM MG132, 10 μM Q1, or 10 μM Q3 prior to stimulation with 20 ng/mL recombinant human TNF (PeproTech; Rocky Hill, NJ, USA). At the end of the stimulation, the medium was removed and cells were fixed with 70% (*v*/*v*) ethanol in water for 20 min at room temperature. Samples were rinsed once with PBS. Endogenous peroxidase was blocked by incubation in 3% (*v*/*v*) hydrogen peroxide (Fisher UK; Loughborough, UK) in PBS for 10 min, followed by three 5 min washes in PBS. Samples were treated with blocking solution (goat anti-rabbit ImmPRESS Polymer Kit—Vector Laboratories; Newark, CA, USA) for 1 h at room temperature, followed by overnight incubation at 4 °C with a rabbit anti-p65/RelA primary antibody (clone D14E12; #8242 Cell Signaling Technology Inc., Danvers, MA 01923, USA) at 1:800 dilution in a blocking solution. The following day, samples were washed three times each for 5 min in PBS, followed by incubation with the secondary antibody (goat anti-rabbit ImmPRESS Polymer Kit; Vector Laboratories) for 1 h at room temperature. Samples were again washed three times each for 5 min in PBS, and the signal was developed using the SIGMAFAST 3,3′-diaminobenzidine (DAB) tablets (Sigma-Aldrich, Poole, UK) to visualize peroxidase activity. The reaction was terminated with tap water. The samples were then stained in hematoxylin (Sigma-Aldrich, Poole, UK) for 4 min, rinsed for 5 min in tap water, and were then mounted in dibutylphthalate polystyrene xylene (DPX) mounting media (Sigma-Aldrich, Poole, UK). Images were taken using a Leica DM LA microscope (Leica Microsystems UK Ltd.; Milton Keynes, UK) at 100× magnification.

A quantitative image analysis to determine TNF-induced p65 nuclear localization from immunocytochemistry and the impact of quinolines Q1 and Q3 was performed using ImageJ software 1.54k by two methods; (i) utilizing the multi-point tool feature to count cells positive for p65 nuclear staining and (ii) digital image analysis with the IHC profiler plugin, as per [40]. Analyses were conducted independently by two researchers. Full details of the image analysis can be found in Supplementary Information File S1.

### 2.7. RNA Extraction and qPCR

RNA extraction and purification from cells were performed using the RNeasy mini kit (Qiagen; Manchester, UK). Purified RNA was reverse transcribed using the High-Capacity RNA-to-cDNA Kit (Applied Biosystems; Paisley, UK), and cDNA was stored at −20 °C. Real-time quantitative PCR (qPCR) was performed using 50 ng total cDNA in 96-well plates (Roche, Burgess Hill, UK) with Taqman Fast advanced master mix (Applied Biosystems, Foster City, CA 94404, USA) and Taqman Gene Expression Assay probes (Applied Biosystems, Foster City, CA 94404, USA), using a qPCR LightCycler 480 (Roche). The Taqman Gene Expression Assay probes used were *TNF* (Hs04404410_s1), *luciferase* (Mr03987587_mr), and results normalized to *GAPDH* (Hs02786624_g1). Conditions for qPCR were as follows: one cycle of 120 s at 50 °C, 20 s at 95 °C; 40 cycles of 3 s at 95 °C, 30 s at 60 °C, and 20 s at 60 °C; one cycle of 120 s at 72 °C and 30 s at 60 °C, as described previously [41]. Cp values were calculated from a 2nd derivative analysis and the relative quantification was calculated using the 2^−ΔΔCT^ method [42].

### 2.8. In Silico Analysis

#### 2.8.1. Molecular Docking

In this study, we used the software AutoDock 4.2 [43] to perform blind docking (to check the whole protein for potential ligand binding sites) on the murine NF-κB heterodimer p50-p65. The Protein Data Bank ID of the crystal structure used is 2I9T (PDB ID: 2I9T; www.rcsb.org/, accessed on 13 July 2024). The experimental procedure was performed as per the AutoDock documentation (https://autodock.scripps.edu/documentation/documentation/, accessed on 13 July 2024). To achieve maximum coverage of NF-κB and the NF-κB-DNA complex during blind docking, only the grid box size was altered beyond the default values of the AutoDock software 4.2.6. Specifically, the number of points in x, y, and z dimensions were changed from the default value of 40 to the maximum value of 126. Spacing was kept at the default value of 0.375 Å and the grid box center coordinates were also kept at the default values, where x = 31.359, y = 23.023, and z = 37.647. The Lamarckian algorithm was used for setting docking parameters, where the top 10 binding poses were retrieved for each ligand.

#### 2.8.2. Molecular Dynamics

Simulations were performed using Desmond software 2024-4 [44] on NF-κΒ-ligand complexes that had the lowest binding energies according to the docking results (meaning the most stable complexes). The conditions of the simulations were 100 ns for the simulation time and water as a solvent at 310 K (37 °C) to simulate physiological conditions. The force field used was the OPLS_2005, as described [45].

#### 2.8.3. Molecular Mechanics/Generalized Born Surface Area (MM/GBSA) Calculations

MM/GBSA was employed to assess the stability of protein–ligand complexes by calculating the free binding energy [46]. The MM/GBSA approach was applied to complex structures using the Prime module within Maestro software 2024-4. The three most statistically significant ligand–protein complexes, identified from an MD trajectory cluster analysis, were subjected to MM/GBSA calculations. The VSGB solvation model, which provides a realistic solvation parameterization, and the OPLS-2005 force field was used to account for protein flexibility. The binding energy was determined using the following equation: ΔG_bind = E_complex (minimized) − E_ligand (minimized) − E_receptor (minimized).

### 2.9. Statistical Analysis

Data are presented as the mean ± standard error of the mean (SEM). Analyses were performed with Prism 8.0 software (GraphPad Software Inc.; San Diego, CA, USA). Pretests were performed to evaluate for normality and equality of variances. Statistical testing involved either parametric one-way analysis of variance (ANOVA) followed by Tukey’s post hoc comparison test or the non-parametric Kruskal–Wallis test to determine significant differences between multiple treatment groups. A *p*-value of less than 0.05 (*p* < 0.05) was considered statistically significant.

## 3. Results

### 3.1. Successful Synthesis of 4-Hydroxy-N’-(4-Methoxybenzylidene)-1-Methyl-2-Oxo-1,2-Dihydroquinoline-3-Carbohydrazide (Q3)

The quinoline compound **3** (Q3) was successfully synthesized in the lab in a high yield (0.32 g, 90% yield), was collected by filtration, then washed with water. mp: 238 °C; LCMS (ESI): m/z 352.10 [M+H]^+^; ^1^H-NMR (500 MHz, CDCl_3_): *δ* 3.73 (s, 3H, N-CH_3_), 3.86 (s, 3H, O-CH_3_), 6.94 (d, *J* = 8.6 Hz, 2H, 4-methoxybenzylidene H3 and H5), 7.35 (t, J = 7.6 Hz, 1H, quinoline H7), 7.40 (d, J = 8.6 Hz, 1H, quinoline H8), 7.71–7.77 (m, 3H, quinoline H6 and 4-methoxybenzylidene H2 and H6), 8.15 (s, 1H, = CH), 8.26 (d, J = 7.9 Hz, 1H, quinoline H5), 12.06 (s, 1H, NH), 13.29 (s, 1H, OH). ^13^C-NMR (125 MHz, CDCl_3_): *δ* 29.49, 55.55, 96.63, 114.36, 114.53, 116.37, 122.89, 125.82, 126.32, 129.79, 134.30, 140.11, 150.21, 161.98, 162.70, 167.81, 172.65; anal. calcd. for C_19_H_17_N_3_O_4_: C, 64.95; H, 4.88; N, 11.96; found: C, 64.65; H, 4.55; N, 11.87. ^1^H-NMR and ^13^C-NMR data of the newly synthesized analog Q3 are presented in Supplementary Information File S2.

### 3.2. Luciferase Assay Condition Set Up

Initial experiments were performed to define the conditions for the luciferase assay, in which the initial number of cells per well (5 to 20 thousand cells), the TNF dose (10 to 40 ng/mL), and the time were tested (1 to 3 h); see Supplementary Information File S3. Based on those data, 2 × 10^4^ cells/well and stimulation with 20 ng/mL TNF for 3 h were suggested as the optimal conditions for subsequent experiments.

**Figure 2:**
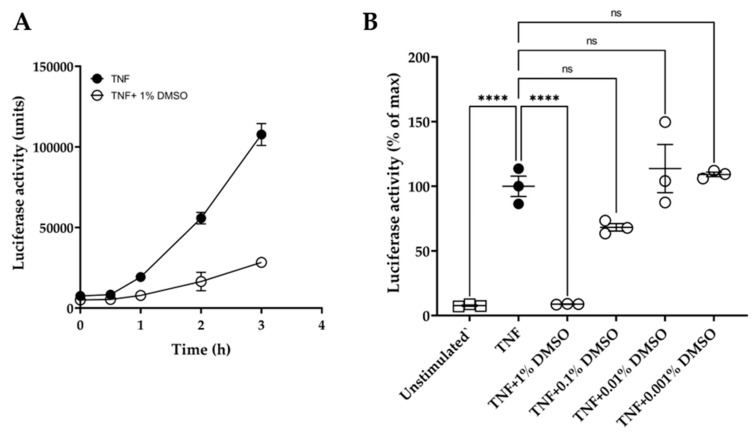
Effect of DMSO on HeLa/NF-κB/luciferase reporter assay. (**A**) Effect of 1% (*v*/*v*) DMSO (o) on TNF-induced luciferase activity over time: TNF treatment at 20 ng/mL (●). (**B**) Dose effect of DMSO dilutions, tested at 1%, 0.1%, 0.01%, and 0.001% (*v*/*v*) in medium, on TNF-induced luciferase activity after 3 h compared to unstimulated controls (□). Data are expressed relative to TNF-stimulated cells (100%). One-way ANOVA was performed, followed by Tukey’s post hoc multiple comparisons of treatment means. Significant differences to TNF-induced cells, **** *p* < 0.0001 (*n* = 3). Non-significant, ns. Data are presented as representative of two independent experiments.

In addition, the quinolines Q1 and Q3 were soluble only in DMSO and not in water or ethanol. DMSO has been reported to affect macromolecules and may interfere in experimental outcomes [47]. Hence, we conducted initial experiments to examine the effect of DMSO on the luciferase reporter assay over time. We showed that 1% (*v*/*v*) DMSO caused a significant inhibition of luciferase activity, with over 70% suppression of the luminescence signal at 3 h (Figure 2A). Moreover, various dilutions of DMSO were tested on TNF-induced luciferase at 3 h (Figure 2B). DMSO did not significantly affect the luciferase assay at dilutions ≥ 1:1000.

**Figure 3:**
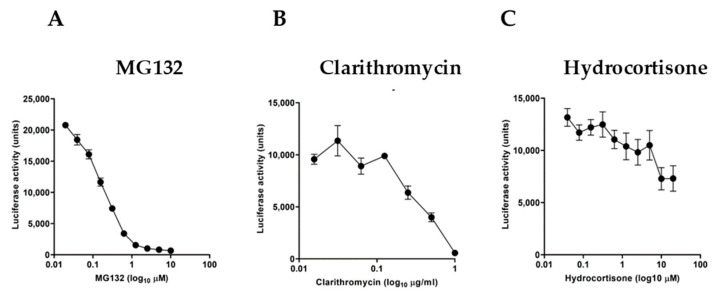
Effect of known NF-κB inhibitors on TNF-stimulated HeLa/NF-κB-Luc cell reporter assay. TNF treatment (at 20 ng/mL, for 3 h) of cells pretreated for 20 min with (**A**) proteasome inhibitor MG132, (**B**) macrolide antibiotic clarithromycin, and (**C**) glucocorticoid steroid hydrocortisone (*n* = 3). Data are presented as representative of three independent experiments.

We further examined the sensitivity of the experimental reporter system by using specific molecules known to act as NF-κB inhibitors. For this purpose, we used the proteasome inhibitor MG132 (Figure 3A), the macrolide antibiotic clarithromycin (Figure 3B), and hydrocortisone (Figure 3C). Serial dilutions of these inhibitors in TNF-stimulated cultures for 3 h showed a differential inhibitory effect with a half-maximal inhibitory concentration (IC50) of 0.1578 μM for MG132, 0.4518 μg/mL for clarithromycin, and 0.8722 μM for hydrocortisone, respectively.

### 3.3. Quinoline Q3 Inhibits NF-κB Activation in TNF-Induced HeLa/NF-κB-Luc Cells

**Figure 4:**
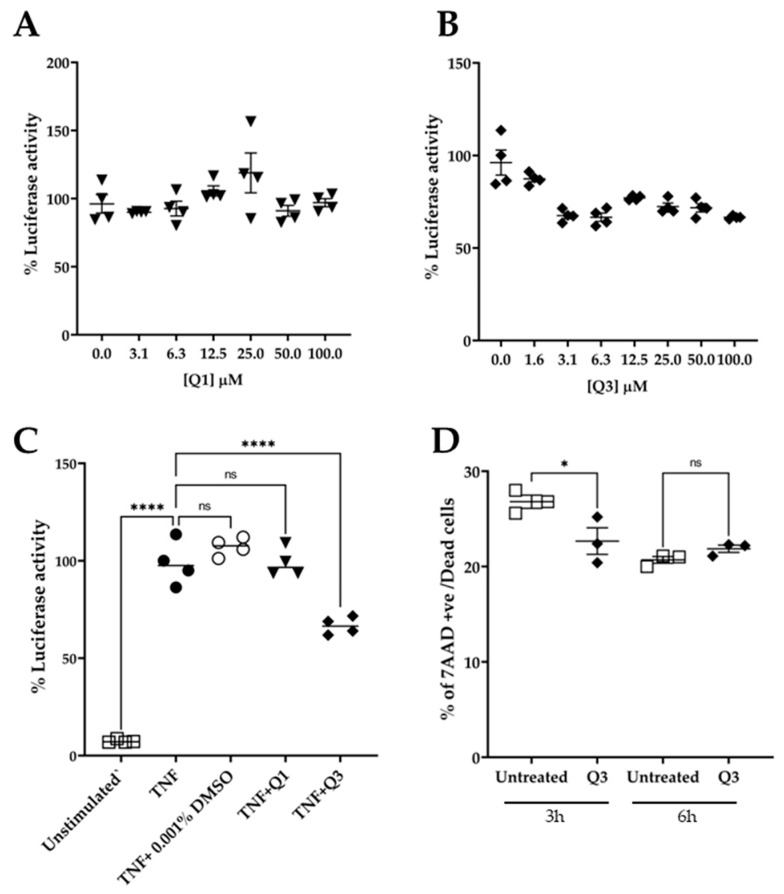
Effect of quinolines Q1 and Q3 on TNF-induced luciferase activity in HeLa/NF-κB-Luc reporter cell line. (**A**) Serial dilutions of Q1 (▼) and (**B**) Q3 (⬪) and (**C**) the effect of Q1 and Q3 at final concentration of 5 μM on luciferase activity in TNF-stimulated cells (●) compared to unstimulated controls (□); *n* = 4. (**D**) Cell viability, as assessed by flow cytometry analysis, of HeLa/NF-κB-Luc cells in the absence or presence of 5 μM Q3 for 3 h and 6 h (*n* = 3). One-way ANOVA was performed, followed by Tukey’s post hoc multiple comparisons of treatment means. Significant differences, * *p* < 0.05 and **** *p* < 0.0001. Non-significant, ns. Data are representative of at least three independent experiments.

To test the effect of quinolines Q1 and Q3 on NF-κB activation, serial dilutions in medium were freshly made from the stock solution prior to addition. The cultures were pre-incubated in the presence of quinolines for 20 min, followed by an addition of 20 ng/mL TNF for 3 h. At the end of the stimulation, the cultures were processed and the luciferase activity was measured. As shown in Figure 4A, pretreatment with Q1 had no impact on the TNF-induced luciferase signal. Conversely, the novel quinoline Q3 significantly inhibited the luminescence signal by approximately 40% compared to TNF alone, at a low concentration of 3 μM. Based on these data, we selected to use Q3 at 5 μM in all subsequent experiments (Figure 4C).

An important factor in studies with potential biological inhibitors is their potential to impact cell viability. We therefore performed experiments in which HeLa/NF-κB-Luc cells were either left untreated or were incubated in the presence of 5 μM Q3 for a time course of up to 6 h (Figure 4D). The cells were harvested, stained with 7AAD, and analyzed by flow cytometry. The results showed that Q3 had no effect on cell viability and did not cause any cell death throughout the duration of the 6 h experiment.

**Figure 5:**
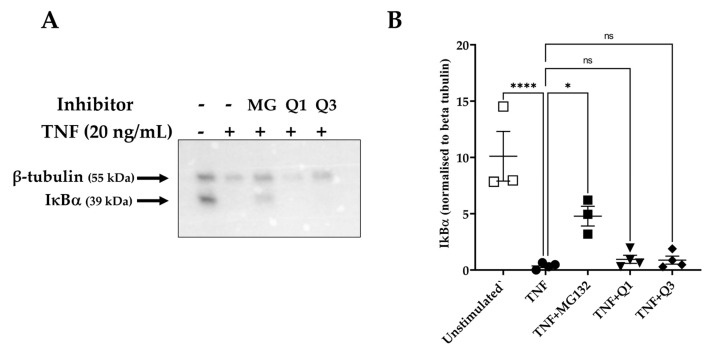
IκBα degradation as assessed by immunoblot analysis in TNF-induced HeLa/NF-κB-Luc reporter cells. HeLa/NF-κB-Luc cells were either left unstimulated (*n* = 3) or stimulated with 20 ng/mL TNF (*n* = 4) for 20 min in the absence or presence of 40 μM MG132 (MG) and 10 μM Q1 or Q3 (all *n* = 4). (**A**) RIPA cell lysates were analyzed with SDS-PAGE, transferred to a PVDF membrane, and levels of IκBα and β-tubulin were each detected by an immunoblot analysis using specific antibodies. Immunoblots are representative of four experiments. All immunoblot experiments can be reviewed in Supplementary Information File S4. (**B**) A quantification analysis of the IκBα bands normalized against β-tubulin levels was performed. One-way ANOVA followed by Tukey’s post hoc multiple comparisons test revealed significant differences. * *p* < 0.05, **** *p* < 0.0001. Non-significant, ns.

The activation of the NF-κB pathway is characterized by the degradation of specific cytoplasmic inhibitors, such as the inhibitor of NF-κB protein alpha (IκBα), and the release of the NF-κB dimers that can then translocate to the nucleus and regulate transcription. Having established that Q3 can inhibit NF-κB-dependent luciferase activity, we then examined whether this effect might be due to interference in upstream events, such as the degradation of IκBα. HeLa/NF-κB-Luc cells were left untreated or stimulated with 20 ng/mL TNF for 20 min, and cell lysates were used to detect levels of IκBα. As shown in Figure 5A,B, TNF markedly induced IκBα degradation within 20 min of stimulation, based on previous studies [48]. However, neither Q1 nor Q3 pretreatment blocked this process. MG132-pretreated cells were used as positive inhibitor control, in which proteasome-dependent IκBα degradation was seen to be inhibited (Figure 5B).

**Figure 6:**
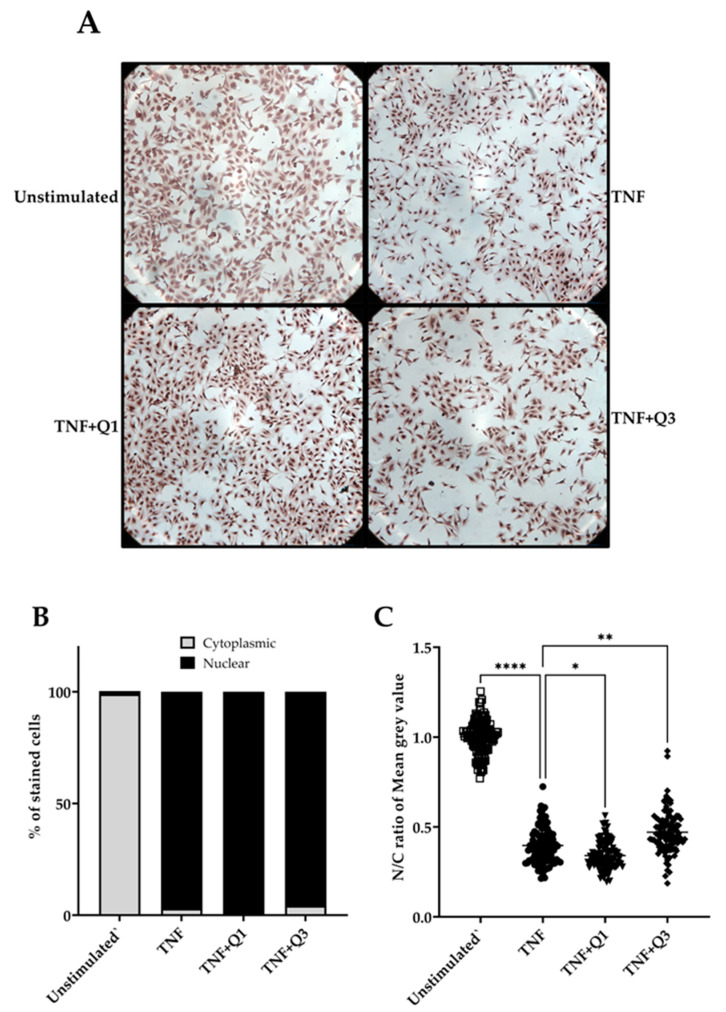
The impact of quinolines Q1 and Q3 on p65/NF-κB nuclear translocation in TNF-induced HeLa/NF-κB-Luc reporter cells. (**A**) Immunocytochemistry images for p65/NF-κB in HeLa/NF-κB-Luc cells left untreated or stimulated with 20 ng/mL TNF for 1 h in the absence or presence of Q1 or Q3. Images are representative of 2 independent experiments, with 3 full field views (100x magnification) per condition for each experiment. Higher-power images can be seen in Supplementary Information File S1. (**B**,**C**) A quantitative analysis to determine TNF-induced p65 nuclear localization (●) compared to unstimulated controls (□) from the immunocytochemistry images and the impact of quinolines Q1 (▼) and Q3 (⬪), performed using ImageJ software 1.54k. Images were analyzed by two approaches. (**B**) Cells showing p65 nuclear staining were counted using the multi-point tool feature in ImageJ; a total of 1000 cells were independently counted per condition by two researchers. Data are illustrated for p65 nuclear (black bars) or cytoplasmic staining (gray bars). (**C**) A digital image analysis was performed using the IHC profiler plugin. Nuclear and cytoplasmic staining, expressed as mean gray value, was measured in cells (*n* = 100, randomly selected across 6 full images for each treatment group) and the nuclear/cytoplasmic (N/C) ratio was calculated. The pixel intensity values for any color ranges from 0 to 255, wherein 0 represents the darkest shade and 255 represents the lightest shade of the color. Therefore, a reduction in the N/C ratio reflects higher amounts of p65 localized within the nucleus. Significant differences, **** *p* < 0.0001, ** *p* < 0.01, and * *p* < 0.05 (Kruskal–Wallis test).

Following quantitative immunocytochemistry image analysis, we observed a significant increase in p65/NF-κB localization following treatment with 20 ng/mL TNF for 1h compared to unstimulated controls (Figure 6A–C). Image analysis based only on positive p65 nuclear staining appeared to show little impact of both quinolines on TNF-induced p65/NF-κB nuclear translocation (Figure 6B). However, a detailed digital analysis of both nuclear and cytoplasmic staining using the IHC profiler in ImageJ demonstrated that Q1 moderately enhances the TNF-induced p65 nuclear signal (*p* < 0.05; Kruskal–Wallis test), whereas, conversely, Q3 was seen to interfere with but not block TNF-induced p65/NF-κB nuclear translocation (*p* < 0.001); see Figure 6C.

**Figure 7:**
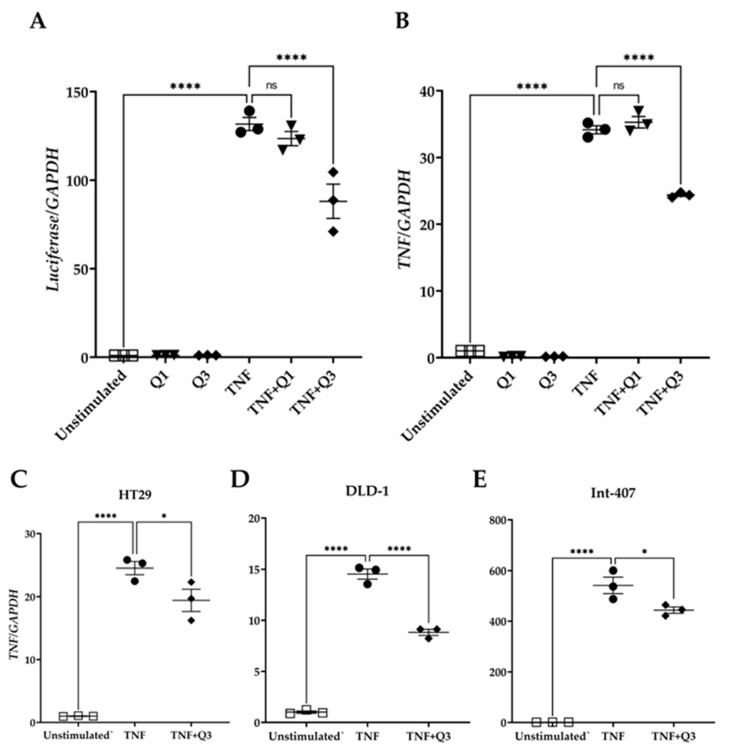
Effect of quinolines Q1 and Q3 on TNF-induced gene expression. Total RNA was isolated from cells either left unstimulated or stimulated with 20 ng/mL TNF for 3 h in the absence or presence of 5 μM Q1 or Q3. Following reverse transcription, a quantification of mRNA abundance was performed by qPCR for (**A**) *luciferase* and (**B**) *TNF* in HeLa/NF-κB-Luc reporter cells. (**C**–**E**) *TNF* gene expression in colorectal/intestinal cell-lines (**C**) HT-29, (**D**) DLD-1, and (**E**) Int-407 cells following TNF stimulation in the absence or presence of Q3. One-way ANOVA was performed, followed by Tukey’s post hoc multiple comparisons of treatment means. Significant differences, * *p* < 0.05, **** *p* < 0.0001. Non-significant, ns. Data are representative of three independent experiments.

To examine whether Q3 could directly affect the NF-κB transcriptional activity, HeLa/NF-κB-Luc cells were cultured in the absence or presence of the quinolines Q1 and Q3 and were either left unstimulated or they were stimulated with 20 ng/mL TNF for 3h. Total RNA was isolated followed by qPCR for *luciferase* and the *TNF* transcript, a known target downstream of the canonical NF-κB pathway. The quinolines alone had neither an impact on *luciferase* nor on *TNF* mRNA abundance (Figure 7). TNF treatment induced an increased expression of *luciferase* gene transcription by 131.7 ± 3.73-fold (Figure 7A). Q1 did not interfere with transcription of this gene (123.48 ± 3.97-fold), whereas Q3 suppressed *luciferase* gene transcription by approximately 40% (88.07 ± 9.68-fold; see Figure 7A). Similarly, TNF treatment induced *TNF* gene expression by 34.15 ± 0.61-fold compared to unstimulated control cells (Figure 7B). Q1 did not interfere with *TNF* gene transcription (35.28 ± 0.86-fold change), whereas Q3 inhibited *TNF* transcription by approximately 35% (24.38 ± 0.21-fold change; Figure 7B). Quinoline Q3 also reduced TNF-induced *TNF* expression in colorectal/intestinal cell lines HT29, DLD-1, and Int-407 (20%, 40%, and 20% reductions, respectively); all *p* < 0.001, *n* = 3 (Figure 7C–E).

### 3.4. Molecular Docking and Molecular Dynamics Analysis

To test the possibility that Q3 exerts its effects by directly interfering with NF-κB itself or the whole protein–DNA complex, we proceeded to molecular docking studies to examine the plausibility of this hypothesis and to potentially reveal a mechanism that explains the effects of Q3. Molecular docking calculates the binding energy (ΔG) of a ligand to its target. The more negative the binding energy, the stronger the binding.

Table 1 shows the top ten binding energies obtained from the docking experiments, indicating the strongest molecular interactions. When docked to NF-κB alone, Q1 and Q3 appear to have similar binding energies; however, when docked to the whole NF-κB-DNA complex, Q3 binds tighter to the complex than Q1. The fact that the docking analysis revealed favorable binding energy for Q3 implies that Q3 inhibits the activation of p65/NF-κB, possibly by interfering with DNA binding. AutoDock docking simulations for genistein-NF-κB acted as a control dataset [49].

While molecular docking provides an initial prediction of ligand–protein binding affinity by calculating the binding energy, it only captures a static ‘snapshot’ of the interaction. To address this limitation and verify the stability of this interaction, we employed molecular dynamics simulations. This approach allows for the observation of the ligand—protein complex over time, providing a more comprehensive understanding of the interaction stability. In addition, the interaction can be studied under biologically relevant conditions (in water and at 37 °C to simulate physiological conditions).

**Figure 8:**
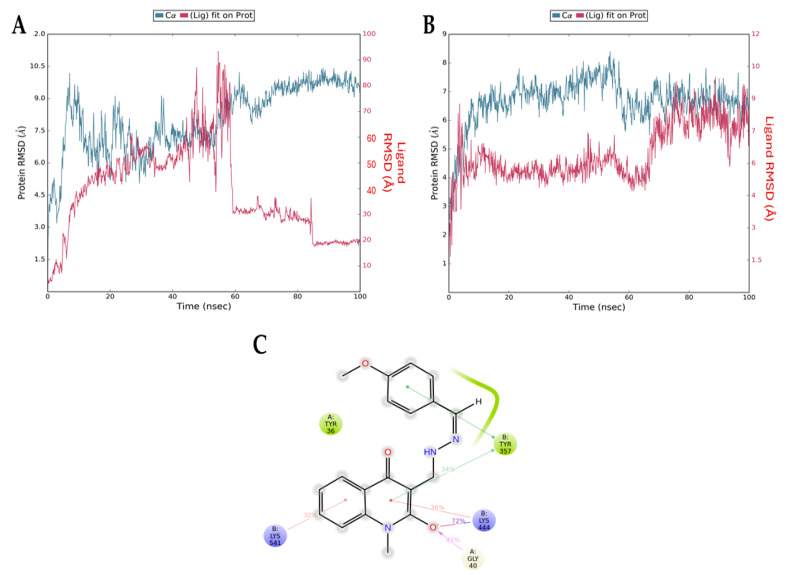
RMSD graphs from molecular dynamics simulations for the NF-κB backbone (blue) and the NF-κB/ligand complex (red) for (**A**) Q1 and (**B**) Q3. The downward fluctuation in red close to the 60 ns timepoint indicates that Q1 diffuses away from NF-κB and there is no complex formation. In the second graph, the overlapping blue and red lines indicate the formation of a stable complex. (**C**) Interactions of Q3 and NF-κB residues formed during MD simulation. The Gly40 of chain A (p65 monomer) and the Tyr357 of chain B (p50 monomer) are found in the RHD of NF-κB, responsible for p50–p65 dimerization, nuclear localization, and DNA binding.

Figure 8 presents the root mean squared deviation (RMSD) graphs of Q1 (control, Figure 8A) and Q3 (Figure 8B) with NF-κB. The RMSD measures the average change in the displacement of atoms. The blue line refers to the protein (NF-κB) backbone, meaning the alpha carbon atoms of its amino acids, and the red line shows how stable the ligand is with respect to the protein and its binding pocket. The downward fluctuation of the red line is an indication that Q1 has diffused away from NF-κB, contradictory to the results of the docking analysis, which indicated a stable interaction. The opposite result can be seen in Figure 8B, where both lines overlap after the 60 ns timepoint. This overlap shows that the protein backbone (blue) and the ligand bound to the protein (red) have similar RMSD values (i.e., atom displacement), which indicates a stable interaction. This is consistent with the docking result that we acquired for the Q3 molecule. Additionally, Figure 8C shows the interactions of Q3 with key amino acid Gly40 of p65 and Tyr357 of p50 NF-κB subunits that form the most studied heterodimer of the canonical pathway.

### 3.5. MM/GBSA Calculations

MM/GBSA calculations were performed on the most statistically significant ‘protein–ligand’ complexes identified through clustering of the Desmond simulation trajectories. Based on the ΔG_bind values shown, Q3 binds more favorably to NF-κB. For NF-κB, the main cluster of Q1 exhibits a ΔG_bind of −30.64 kcal/mol, while for Q3, the ΔG_bind is comparatively lower at −39.70 kcal/mol.

## 4. Discussion

NF-κB is a key transcription factor that regulates many biological processes. The dysregulation and hyperactivation of the canonical NF-κB pathway is linked with multiple pathologies including chronic inflammatory diseases such as inflammatory bowel disease [50], rheumatoid disease [51], and cancer [52]. We showed previously that in human blood-derived macrophages, the endogenous NF-κB activation profile can segregate IBD patients, as it shows hypoactivation in ulcerative colitis patients and hyperactivation in Crohn’s disease patients who smoke [53]. These findings render the NF-κB transcription factor an important target for drug design against inflammatory diseases and cancer [21].

Amongst those drugs designed to target the NF-κB pathway, only glucocorticoids and peroxisome proliferator-activated receptor (PPAR) agonists were shown to interfere in the DNA-binding activity of this transcription factor, as summarized by Quo et al. recently [21]. Quinolines have been previously reported to inhibit the NF-κB pathway [31,32]. Similarly, Xu et al. reported analogs of quinazoline, another class of *N*-based heterocyclic compounds structurally like quinolines, that acted as NF-κB inhibitors by potentially binding to the p50 subunit based on a molecular docking analysis [54]. A previous study used transient transfection of an NF-κB reporter construct and showed that the hydroxyquinoline Clioquinol in combination with zinc enhanced the radiosensitivity of HeLa and MCF-7 cells by inhibiting the NF-κB signaling pathway in vitro [55]. In this study, we used for the first time an NF-κB-specific HeLa transgenic luciferase reporter cell line to test a novel quinoline molecule. We validated the use of the specific cell line by testing known NF-κB inhibitors, such as MG132, clarithromycin, and hydrocortisone. The novel Q3 compound exerted an inhibitory action on the canonical NF-κB pathway compared to the control Q1 molecule, which could be explained by the structure of the molecule, since Q3 has a double bond and is probably more rigid compared to the control compound Q1. Q3 did not interfere with cell viability, but it inhibited TNF-induced activation of the luciferase reporter. This TNF-induced inhibitory activity was not due to the suppression of IκBα degradation. An image analysis showed interference of p65 nuclear translocation by Q3 but not by the Q1 compound. The latter favored p65 nuclear translocation, an observation that was seen in a previous study examining the fluoroquinolone Trovafloxacin that markedly induced TNF- and LPS-induced p65 nuclear translocation [56].

Since quinolines and quinolones act by interfering with DNA-binding enzymes, we decided to investigate through in silico methods whether this inhibitory activity of Q3 could be due to direct binding with NF-κB and interference with its DNA-binding activity. In our experiments, the binding energy of Q3 with the p65/DNA complex was −9.13 kcal/mol, much lower than the control molecule Q1 (−7.80 kcal/mol). Previous docking studies have reported NF-κB interactions with other molecules, including the study of Mukherjee et al., where it was shown that p65 interacts with protein lysine acetyltransferase CBP/p300, an important regulator of gene transcription [57] and genistein, a chemopreventive isoflavonoid known to inhibit the translocation and expression of NF-κB in the nucleus of various breast cancer cell lines [49]. The latter study also employed AutoDock docking simulations for genistein, revealing that it bound to NF-κB with a binding energy of −6.29 kcal/mol, indicating only moderate affinity compared to that seen here for quinolines Q1 and Q3. The genistein interaction was stabilized though by several key hydrogen bonds involving residues Lys52, Ser243, Asp274, and Lys275, and these interactions were thought to potentially inhibit NF-κB activity, which could help in suppressing cancer-related gene expression in breast cancer cells. We identified key interactions of Q3 with Gly40 of the p65 monomer through a hydrogen bond and Tyr357 of the p50 monomer through two different pi–pi stacking interactions. Both of these residues are located within the RHD found in the N-terminal region of NF-κΒ proteins, responsible for p65–p50 dimerization, nuclear localization, and DNA binding [4]. The low binding energy values we observed indicate that direct interaction with NF-κB is plausible. The interactions of Q3 with amino acid residues within the RHD indicate that Q3 could potentially interfere not only with NF-κB DNA-binding activity but also its dimerization.

Based on the in silico analysis and evidence gathered from our in vitro experiments presented here, future research should assess the effects of Q3 on primary cells, such as intestinal stem cell-derived 3D organoid cultures that better resemble the gut epithelium [58]. Moreover, it could be advantageous to aim for modifications that increase the solubility of Q3 in diluents such as ethanol, an approach that will facilitate further experiments in ex vivo and in vivo preclinical models. Verification of the binding of Q3 to NF-κB would also yield great insights for the targeting of NF-κB with small molecules and could be used for ligand-based drug design (LBDD).

## 5. Conclusions

We have synthesized a novel quinoline compound, named Q3, that can effectively inhibit NF-κB/p65 transcriptional activity and interfere with its nuclear translocation in the HeLa/NF-κB-Luc and intestinal epithelial cell lines. The inhibitor Q3 neither blocked IκB degradation nor did it affect cell survival. Docking and molecular dynamics analyses confirmed that Q3 could potentially interfere with transcriptional activity and hence could be potentially developed for further in vivo studies as an NF-κB inhibitor.

## Figures and Tables

**Table 1:** Binding energies (kcal/mol) of Q1 and Q3 with NF-κB and NF-κB-DNA complex.

*Q1+NF-κB*	*Q3+NF-κB*	*Q1+complex*	*Q3+complex*
−6.61	−6.50	−7.80	−9.13
−6.20	−5.62	−7.57	−8.69
−5.63	−5.36	−7.43	−8.49
−5.49	−5.34	−7.43	−8.17
−5.34	−4.74	−7.35	−8.13
−5.31	−4.73	−7.32	−7.56
−5.16	−4.57	−7.32	−7.41
−5.10	−4.49	−7.19	−7.19
−4.92	−4.44	−6.88	−7.13
−4.92	−4.34	−5.93	−7.11

## Data Availability

The original contributions presented in the study are included in the article/Supplementary Material; further inquiries can be directed to the corresponding author/s.

## Supplementary Materials

The following supporting information can be downloaded at: https://www.mdpi.com/article/10.3390/biology13110910/s1, Supplementary Information File S1: Immunocytochemistry image analysis methodology; Supplementary Information File S2: Proton and carbon NMR data; Supplementary Information File S3: Cell density and TNF dose- and time-dependent effect on NF-κB luciferase reporter assay; and Supplementary Information File S4: IκBα degradation as assessed by immunoblot analysis in TNF-induced HeLa/NF-κB-Luc reporter cells.
