# Supplementary material for: A Novel Quinoline Inhibitor of the Canonical NF-κB Transcription Factor Pathway
Source: Biology (Basel). 2024 Nov 7;13(11):910. doi: 10.3390/biology13110910 (PMC11591978; doi:10.3390/biology13110910)
Supplement: Supplementary file 1 [file biology-13-00910-s001.zip › Supplementary Information File S1 - Imaging analysis methods.pdf]

A novel quinoline inhibitor of the canonical NF-κB transcription factor pathway

Panagiotis Ntavaroukas, Konstantinos Michail, Rafaela Tsiakalidou, Eleni Stampouloglou, Katerina Tsiggene, Dimitrios Komiotis, Stella Manta, Nikitas Georgiou, Thomas Mavromoustakos, Danielle Aje, Panagiotis Michael, Barry J. Campbell and Stamatia Papoutsopoulou

Supplementary Information File S1

Immunocytochemistry image analysis methodology.

The ImageJ software (https://imagej.net/ij/) was used to analyze the immunocytochemistry images. This was performed using two approaches:

The first approach utilized the multi-point tool feature in ImageJ to count cells (n = 1000, across 6 full field images per condition) showing p65 nuclear staining, conducted independently by two researchers.

The second, digital image analysis approach used the IHC profiler plugin (Varghese *et al.*, 2014). Here TIFF files were converted to RGB images. The IHC profiler plugin allowed the deconvolution of the RGB image to (i) a hematoxylin image and (ii) a 3,3′-diaminobenzidine (DAB) image. In the DAB image, with the use of the freehand tool, the nucleus and cytoplasm of cells (100 cells randomly selected across 6 images, per condition) were analyzed and the mean grey value (pixels) was measured. Representative images of the digital analysis process for each condition is shown in **Figure S1** (below).

Reference:

Varghese F, Bukhari AB, Malhotra R, De A. IHC Profiler: an open-source plugin for the quantitative evaluation and automated scoring of immunohistochemistry images of human tissue samples. PLoS One. 2014; 9(5): e96801. doi: 10.1371/journal.pone.0096801. PMID: 24802416; PMCID: PMC4011881.

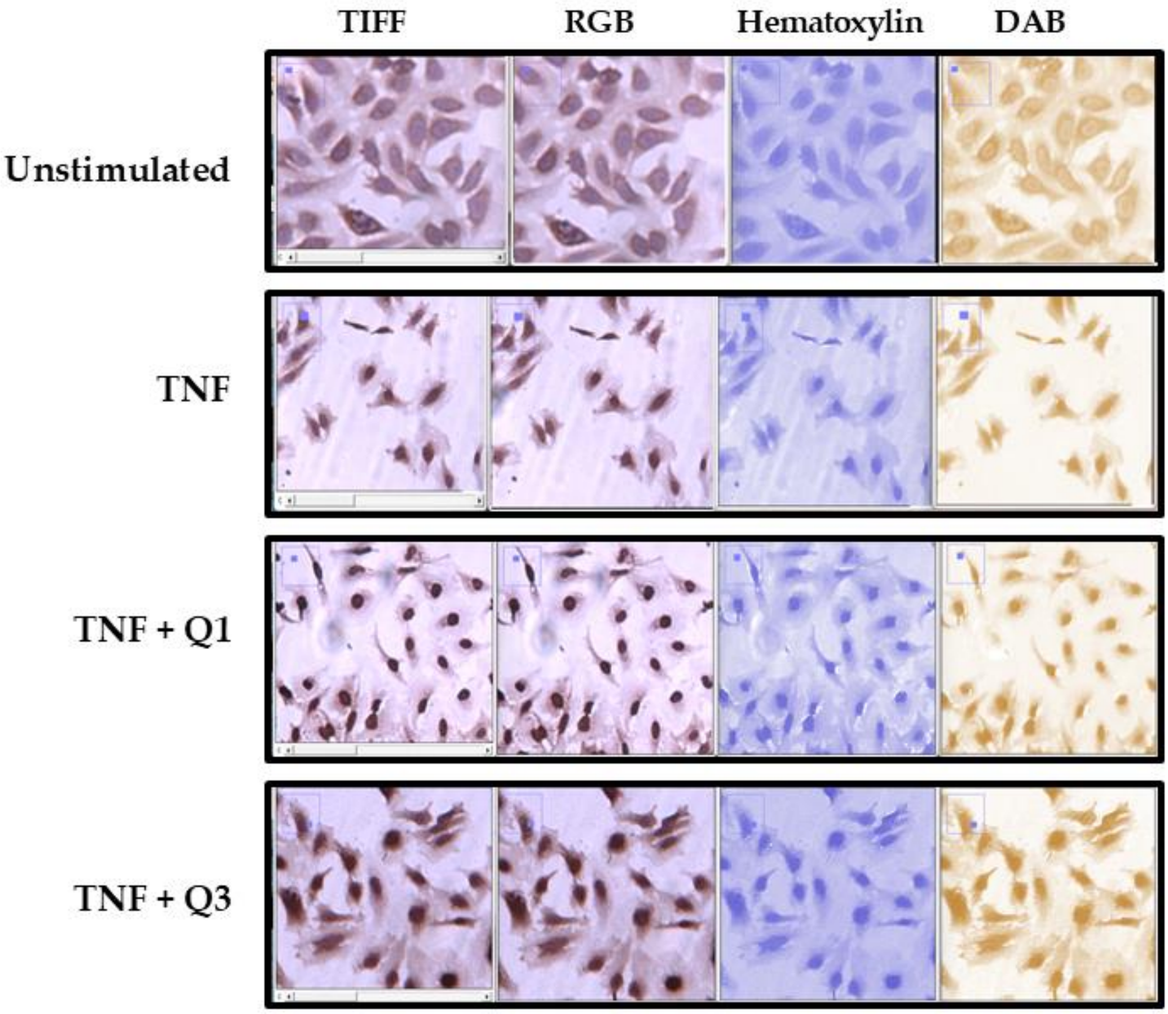

Figure S1. **Digital analysis of p65 stained HeLa-NF-κB-Luc cells using the IHC Profiler in ImageJ.** Quantitative analysis to determine TNF-induced p65 nuclear localization compared to unstimulated controls from the immunocytochemistry images, and the impact of quinolines Q1 and Q3. Images, as TIFF files, were converted to RGB images and deconvolution performed to generate a hematoxylin image and then a 3,3′-diaminobenzidine (DAB) image. Nuclear and cytoplasmic staining, measured in the DAB image was expressed as mean grey value, across 100 randomly selected cells from images for each treatment group. The pixel intensity values for any color ranges from 0 to 255, wherein, 0 represents the darkest shade and 255 represent the lightest shade of the color. Therefore, reduction of the nuclear to cytoplasmic ratio reflects higher amounts of p65 localized within the nucleus. Two independent experiments were performed, with three full field images taken per experiment.
