# Supplementary material for: A Novel Quinoline Inhibitor of the Canonical NF-κB Transcription Factor Pathway
Source: Biology (Basel). 2024 Nov 7;13(11):910. doi: 10.3390/biology13110910 (PMC11591978; doi:10.3390/biology13110910)
Supplement: Supplementary file 1 [file biology-13-00910-s001.zip › Supplementary Information File S2 - Proton and Carbon NMRs.pdf]

A novel quinoline inhibitor of the canonical NF-κB transcription factor pathway

Panagiotis Ntavaroukas, Konstantinos Michail, Rafaela Tsiakalidou, Eleni Stampouloglou, Katerina Tsiggene, Dimitrios Komiotis, Stella Manta, Nikitas Georgiou, Thomas Mavromoustakos, Danielle Aje, Panagiotis Michael, Barry J. Campbell and Stamatia Papoutsopoulou

Supplementary Information File S2

A

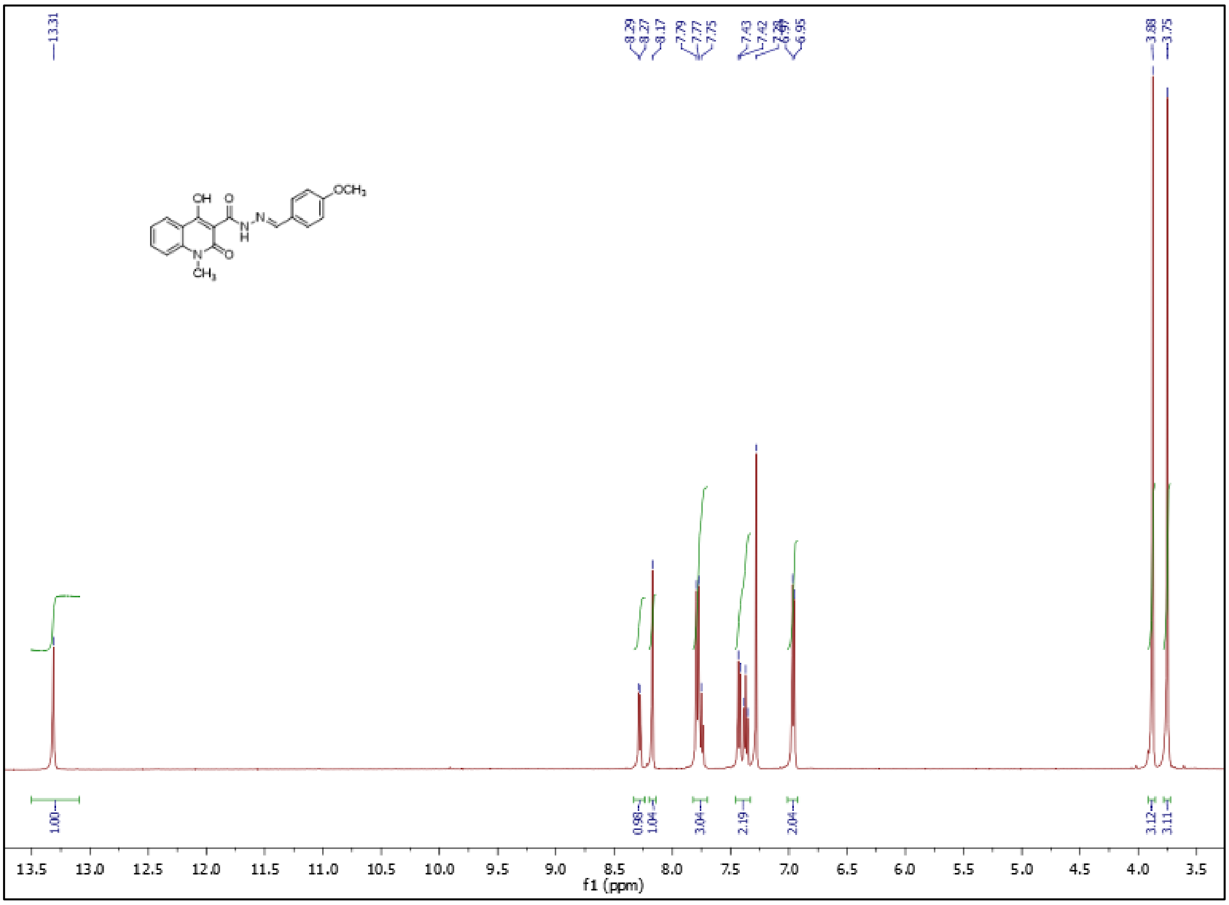

B

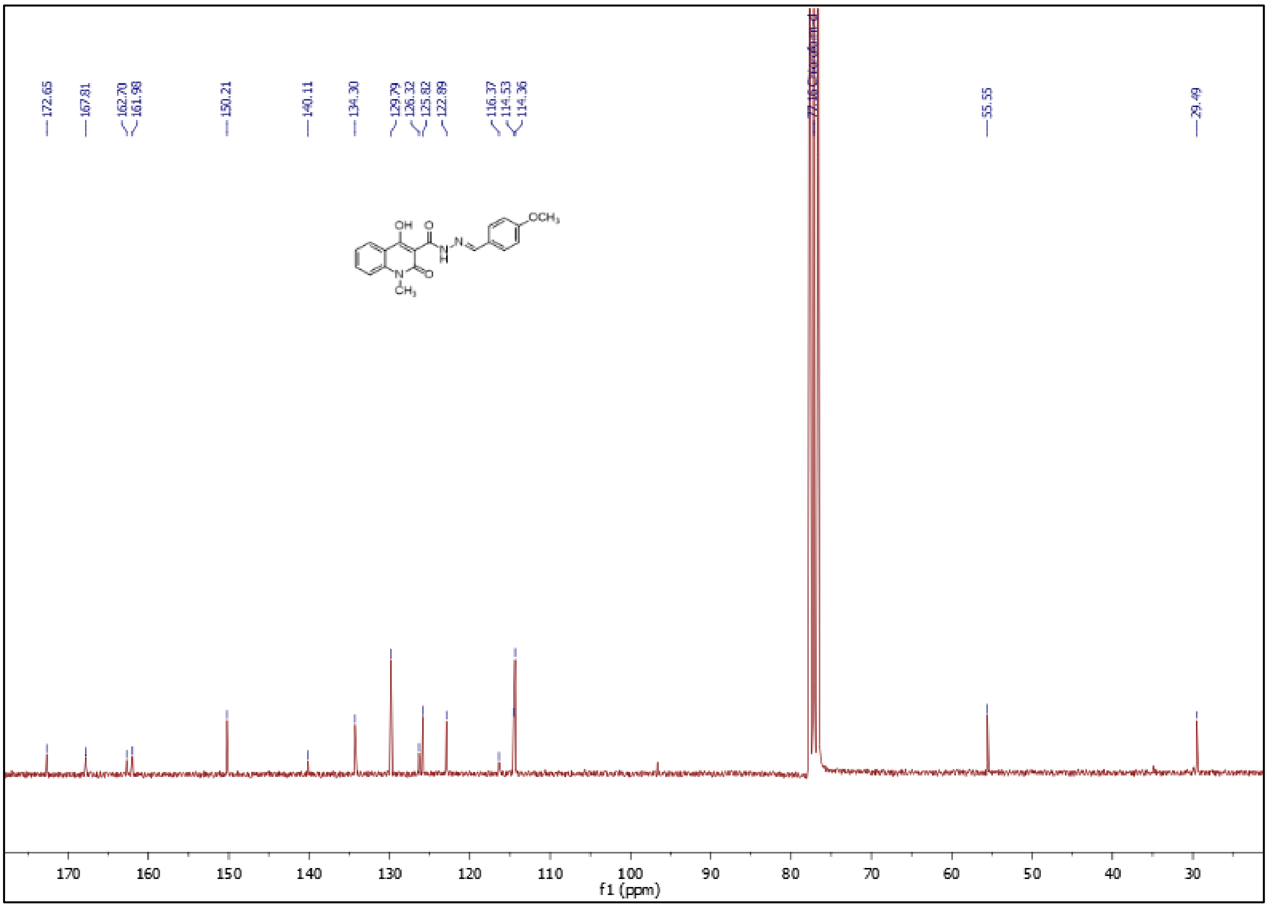

Figure S2: **^{1}H (A) and ^{13}C (B) NMR spectra of quinoline compound Q3**. Melting points were recorded in a DigiMelt MPA 160 Mel-Temp apparatus and are uncorrected. Thin layer chromatography (TLC) was performed on Merck pre-coated 60F254 plates. Reactions were monitored by TLC on silica gel, with detection by UV light (254 nm) or by charring with sulfuric acid. Flash column chromatography was performed using silica gel (240-400 mesh, Merck). ^{1}H and ^{13}C NMR spectra were obtained at ambient temperature using a Bruker Avance 500 spectrometer at 500 and 125 MHz, respectively using deuterated chloroform (chloroform-d; $CDCl_3$) with internal tetramethylsilane (TMS). Chemical shifts (δ) are given in ppm measured downfield from TMS, and spin–spin coupling constants in Hz. Mass spectra were obtained on a ThermoQuestFinnigan AQA Mass Spectrometer (electrospray ionization). All reactions sensitive to oxygen or moisture were carried out under an argon atmosphere.
