# Supplementary material for: A Novel Quinoline Inhibitor of the Canonical NF-κB Transcription Factor Pathway
Source: Biology (Basel). 2024 Nov 7;13(11):910. doi: 10.3390/biology13110910 (PMC11591978; doi:10.3390/biology13110910)
Supplement: Supplementary file 1 [file biology-13-00910-s001.zip › Supplementary Information File S3 - Cell density and TNF dose.pdf]

A novel quinoline inhibitor of the canonical NF-κB transcription factor pathway

Panagiotis Ntavaroukas, Konstantinos Michail, Rafaela Tsiakalidou, Eleni Stampouloglou, Katerina Tsiggene, Dimitrios Komiotis, Stella Manta, Nikitas Georgiou, Thomas Mavromoustakos, Danielle Aje, Panagiotis Michael, Barry J. Campbell and Stamatia Papoutsopoulou

Supplementary Information File S3

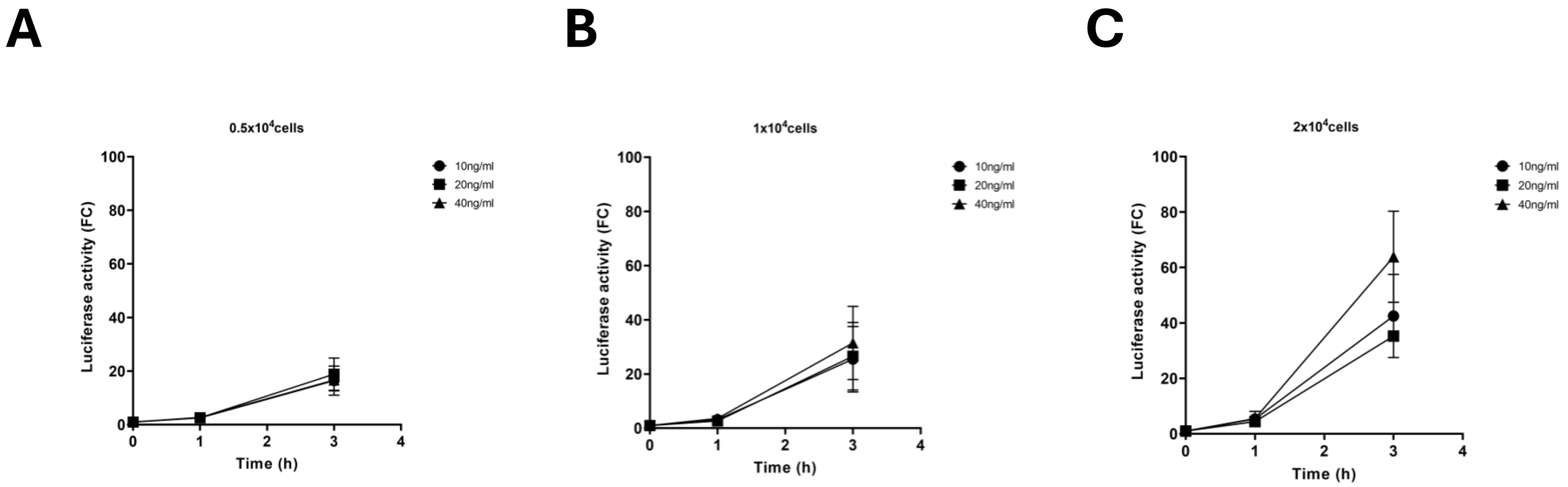

Figure S3: Cell density and TNF dose- and time-dependent effect on the NF-κB luciferase reporter assay. HeLa/NF-κB-Luc cells were seeded to 96 well plates at different densities, (**A**) 0.5 x 10^4, (**B**) 1 x 10^4 and (**C**) 2 x 10^4 cells/well, cultured overnight and were stimulated for up to 3 h with three different doses of recombinant human TNF (10, 20 and 40 ng/mL). At the end of each experiment the cultures were washed with sterile phosphate-buffered saline (PBS), cells lysed with Bright-Glo and analyzed for luminescence signal as described in *2.3 Materials and Methods*.
