# Supplementary material for: A Novel Quinoline Inhibitor of the Canonical NF-κB Transcription Factor Pathway
Source: Biology (Basel). 2024 Nov 7;13(11):910. doi: 10.3390/biology13110910 (PMC11591978; doi:10.3390/biology13110910)
Supplement: Supplementary file 1 [file biology-13-00910-s001.zip › Supplementary Information File S4. Western blots.pdf]

A novel quinoline inhibitor of the canonical NF-κB transcription factor pathway

Panagiotis Ntavaroukas, Konstantinos Michail, Rafaela Tsiakalidou, Eleni Stampouloglou, Katerina Tsiggene, Dimitrios Komiotis, Stella Manta, Nikitas Georgiou, Thomas Mavromoustakos, Danielle Aje, Panagiotis Michael, Barry J. Campbell and Stamatia Papoutsopoulou

Supplementary Information File S4

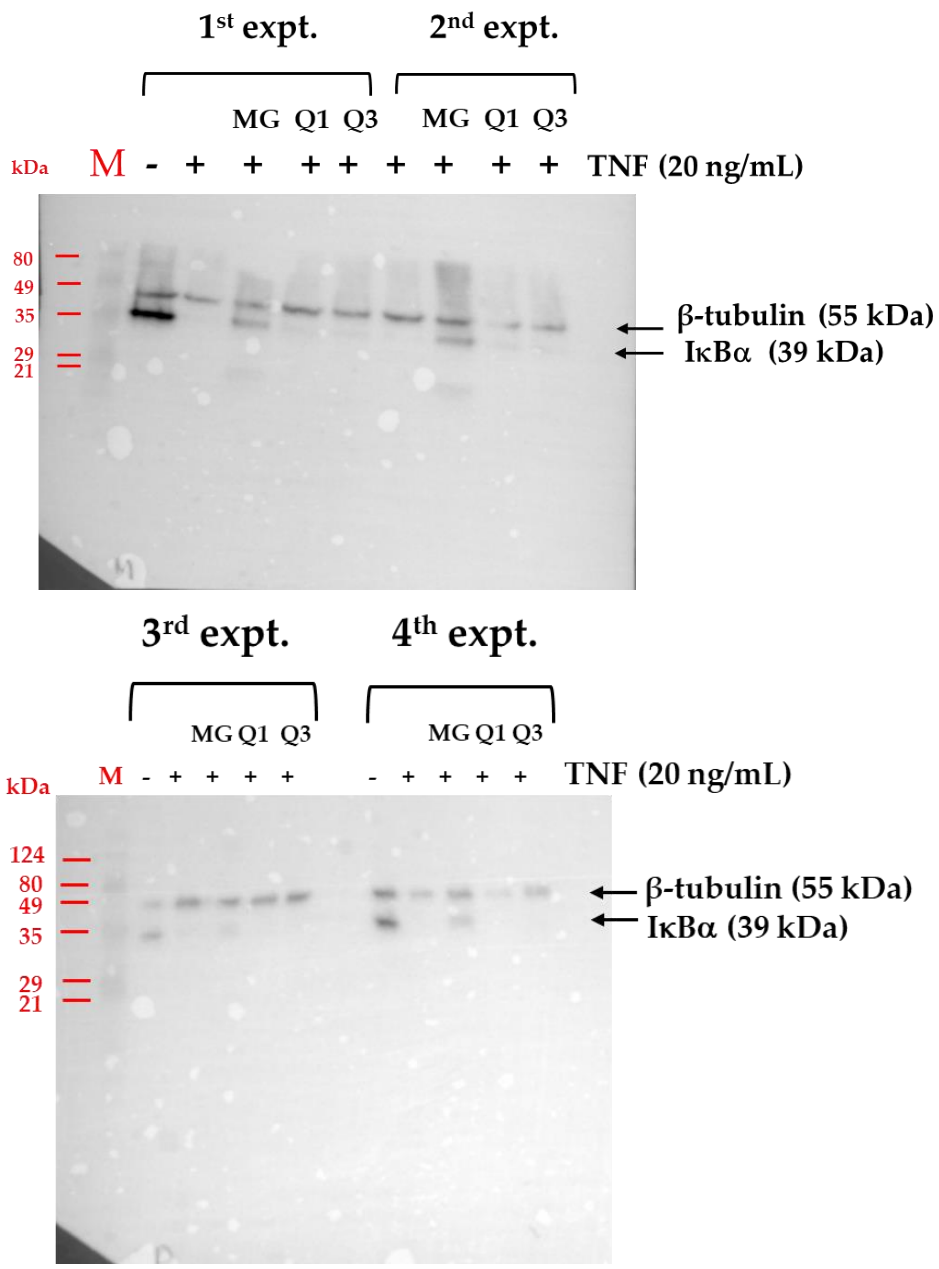

Figure S4: Western blots examining the action of quinolones Q1 and Q3 on TNF-induced degradation of IκBα (NF-κB pathway activation) in HeLa cell lysates.

HeLa/NF-κB-Luc cells were seeded to 96 well plates at different densities were either left untreated or stimulated with 20 ng/mL recombinant human TNF in the absence or presence of 40 μM MG132 (MG), 10 μM Q1 or 10 μM Q3 for 20 min (optimum time to detect IκBα degradation post TNF stimulation, as previously reported by Krappmann and Scheidereit, 1997). Cells were rinsed with PBS and lysed in RIPA lysis buffer and samples were subjected to electrophoresis on SDS-PAGE followed by trans-blot to PVDF membrane. IκBα and β-tubulin levels were probed sequentially using specific antibodies, followed by secondary peroxidase conjugated antibody amplification and detection using enhanced chemiluminescence (ECL) imaging. Full details described in *2.5 Materials and Methods*. Data illustrated is from 4 independent experiments.

Reference:

Krappmann D, Scheidereit C. Regulation of NF-kappa B activity by I kappa B alpha and I kappa B beta stability. Immunobiology 1997; 198(1-3): 3-13. doi: 10.1016/s0171-2985(97)80022-8. PMID: 9442373.
